# Evaluation of communication between physicians and patients in Astana City Hospital №1

**DOI:** 10.5195/cajgh.2013.82

**Published:** 2014-01-24

**Authors:** Anara Zhumadilova, Brett J. Craig, Alexey Tsoy, Alla Gabdrakhmanova, Martin Bobak

**Affiliations:** 1 School of Science and Technology, Nazarbayev University, Astana, Kazakhstan; 2 Astana City Hospital #1, Astana, Kazakhstan; 3 University College London, London, United Kingdom

**Keywords:** doctor-patient interaction, communciation, PPOS

## Abstract

**Introduction:**

Communication between patients and health care providers is important for the effective functioning of health care systems. Miscommunication often stems from discrepancies in expectations of both healthcare professionals and patients due to cultural and historical influences. We investigated the degree to which health care providers (doctors and nurses) and patients in Kazakhstan believe that interaction between doctors and patients should be doctor- or patient-oriented.

**Material and methods:**

We conducted a cross-sectional study of 163 patients and 176 health care providers (71 doctors and 105 nurses) in a general hospital in Astana, Kazakhstan. The subjects completed a structured questionnaire containing the Patient-Practitioner Orientation Scale (PPOS), and scales assessing life and job satisfaction, effort-reward balance of healthcare professionals, and the patients’ perceptions of communication practices.

**Results:**

An overwhelming majority of doctors (81.7%), nurses (88.1%), and patients (92.3%) were doctor-oriented. Among health care providers, PPOS was not associated with age, sex, life and job satisfaction, or effort-reward imbalance. Among patients, PPOS was not associated with age, sex, or specialty of health care provider. However, higher PPOS among patients (indicating preference for patient-oriented interaction) was associated with higher satisfaction with communication with health care providers and, less strongly, with their life satisfaction.

**Conclusion:**

The main finding of this study is the very small proportion of doctors, nurses and patients who believe that interaction should be patient-oriented. These results highlight the necessity of improvement of communication among health care providers towards patient-oriented approach in order to decrease miscommunication with patients. The fact that most patients prefer doctor-oriented interaction may reflect historical stereotypes; educational/information interventions among patients may also be needed.

